# 

**Published:** 1867-10

**Authors:** 


*Apology.*—Some apology is due our readers for a number of typographical errors in the July and August numbers of the Journal. These errors resulted from our not having an opportunity to correct the proof sheets, owing to absence from home during the hot weather.

